# Surface degeneration of W crystal irradiated with low-energy hydrogen ions

**DOI:** 10.1038/srep23738

**Published:** 2016-03-29

**Authors:** Hongyu Fan, Yuwei You, Weiyuan Ni, Qi Yang, Lu Liu, Günther Benstetter, Dongping Liu, Changsong Liu

**Affiliations:** 1 School of Physics and Materials Engineering, Dalian Nationalities University, Dalian 116600, People’s Republic of China; 2 Key Laboratory of Materials Physics, Institute of Solid State Physics, Chinese Academy of Sciences, P. O. Box 1129, Hefei 230031, P. R. China; 3 Faculty of Electrical Engineering and Media Technology, Deggendorf Institute of Technology, Deggendorf 94469, Germany

## Abstract

The damage layer of a W (100) crystal irradiated with 120 eV hydrogen ions at a fluence of up to 1.5 × 10^25^/m^2^ was investigated by scanning electron microscopy and conductive atomic force microscopy (CAFM). The periodic surface degeneration of the W crystal at a surface temperature of 373 K was formed at increasing hydrogen fluence. Observations by CCD camera and CAFM indicate the existence of ultrathin surface layers due to low-energy H irradiation. The W surface layer can contain a high density of nanometer-sized defects, resulting in the thermal instability of W atoms in the surface layer. Our findings suggest that the periodic surface degeneration of the W crystal can be ascribed to the lateral erosion of W surface layers falling off during the low-energy hydrogen irradiation. Our density functional theory calculations confirm the thermal instability of W atoms in the top layer, especially if H atoms are adsorbed on the surface.

The irradiation with energetic particles in DT fusion phase (D + T → α + n (14 MeV)) leads to the degradation of plasma-facing materials in nuclear reactors. Tungsten (W) as one of the promising plasma-facing materials for ITER will be subject to the irradiation by large flux (10^22^–10^24^/m^2^·s) and low-energy (tens of eV to hundreds of eV) helium (He) and hydrogen (H) ions^1^,^2^. It has been widely accepted that He^+^ irradiation can cause serious surface degeneration of the W crystal, such as the formation of voids, wave-like microstructures, and nanostructured fuzz^3^,^4^,^5^,^6^,^7^. He atoms in W crystal grains may cluster in a close-packed arrangement and form He-enriched strips. The formation of wave-like microstructures and nanostructured fuzz can be due to surface sputtering and swelling of He-enriched strips at an elevated temperature^3^. However, the hydrogen retention, surface swelling, bubbles, blister bursting, and exfoliations are related to the low-energy hydrogen irradiation^8^,^9^,^10^,^11^. Hydrogen atoms permeating through W can be trapped at the defects or combine into molecules at voids, which is responsible for the hydrogen retention^12^,^13^,^14^. Blister characteristics and cracking behavior strongly depend on W microstructures and dopant materials^15^. Simulations suggest that the migration-coalescence of the bubbles and their ultrahigh pressure should be the main driving force for the bubble growth, resulting in blister bursting, and exfoliations^16^,^17^. However, the surface erosion or degeneration due to low-energy hydrogen irradiation still remains unclear.

In this study, the observation of surface degeneration of W (100) crystal surfaces irradiated with low-energy hydrogen ions has been performed by using scanning electron microscopy, a CCD camera, and conductive atomic force microscopy (CAFM). Observations show the existence of damage layer due to low-energy hydrogen ion irradiation, resulting in surface degeneration of W crystals. Density functional theory (DFT) calculations are performed to analyze the thermal stability of W atoms in the surface layer of a W (100) crystal.

## Results

**Figure 1:**
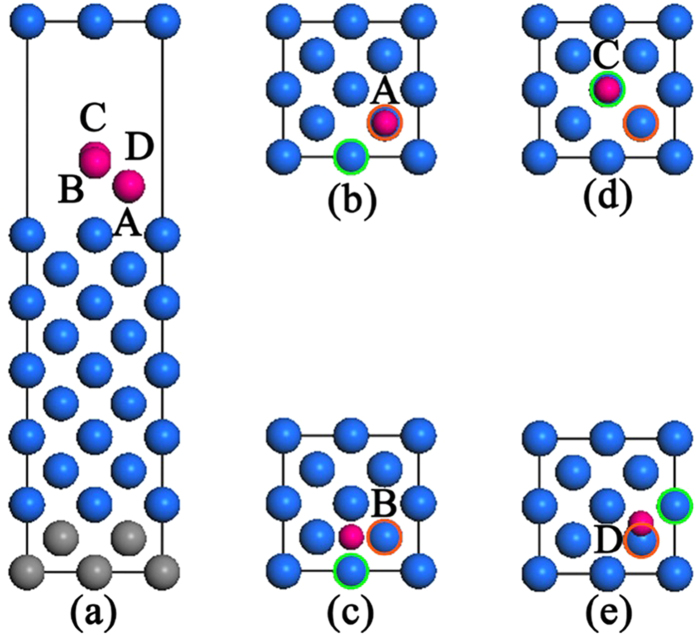
The W (100)-slab with H atoms adsorbed on high-symmetry (A–D) positions (**a**), and their corresponding top views (**b–e**), respectively. The blue balls are W atoms, and the pink balls are H atoms. The grey balls are the W atoms with positions fixed for DFT calculation. The blue balls with green circles are the W atoms escaped from the top layer, and the blue balls with red circles are the W atoms escaped from the second layer.

The thermal stability of the W (100) layers was studied by using DFT calculations. Two cases were proposed for DFT calculations, namely, no H adsorption on the surface and H adsorbing on the surface. Our DFT calculations were performed using the VASP code and ultrasoft pseudopotentials^18^. The generalized gradient approximation was used to deal with exchange–correction energy^19^. A plane-wave basis with energy cutoff of 500 eV was used to expand the electronic wave functions in all the calculations. Structural relaxations were performed until forces on each atom were less than 0.02 eV/Å. For the study of H/W (100), we used a *p* (2 × 2) surface cell and a *k*-point sampling^20^ of 7 × 7 × 1. In the modeling of the W(100) surface, eleven layers of W atoms were used with the bottom two layers fixed at their bulk positions as shown in Fig. 1. We used a 10 Å vacuum layer for the W (100) surface and allowed the H layer together with the unfixed W layers to relax.

**Figure 2:**
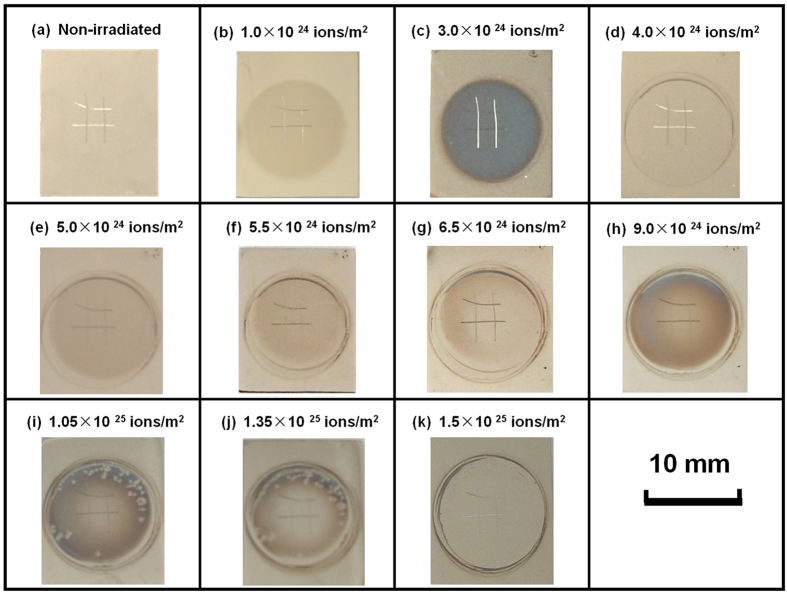
Photographs of W (100) crystal prior to H irradiation (**a**) and irradiated with 120 eV hydrogen ions at fluences of 1.0 × 10^24^–1.5 × 10^25^/m^2^ (**b–k**). This W specimen was marked for *in-situ* observation by a CCD camera and a SEM.

The images of the polycrystalline W specimen exposed to hydrogen fluences ranging from 1.0 × 10^24^ to 1.5 × 10^25^/m^2^ are shown in Fig. 2. A periodic change in the color of the irradiated W is clearly observed while the hydrogen ion fluence increases from 1.0 × 10^24^ to 1.5 × 10^25^/m^2^. During each period, the images initially become dark in color and then turn into light color. The periodic change in color results from an alteration in the microstructures of the W surface layer due to the hydrogen ion irradiation. The dark color of the W surface indicates the formation of a surface layer during the hydrogen ion irradiation. After each period, the light color of the W surface indicates the removal of a surface layer due to the hydrogen ion irradiation. The total hydrogen ion fluence resulting in each periodic change is approximately 4.0 × 10^24^ ions/m^2^.

**Figure 3:**
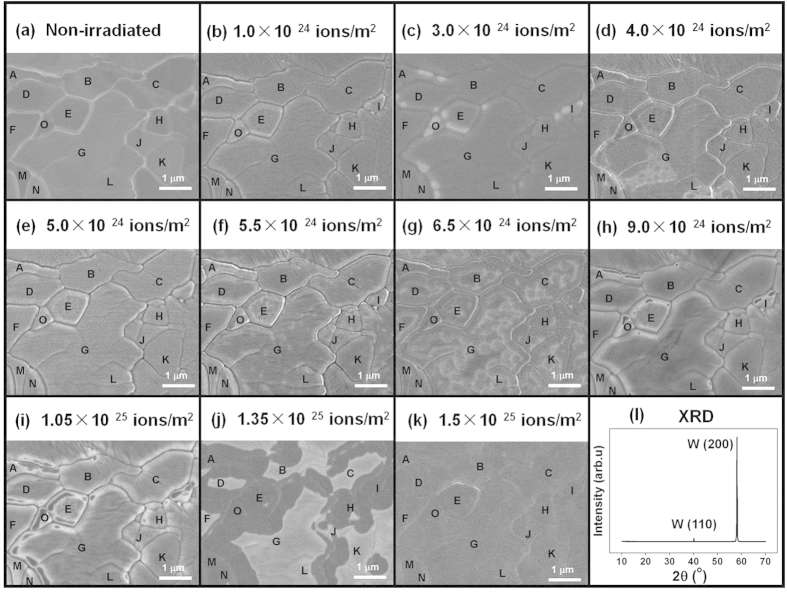
SEM observation for the W (100) crystal prior to H irradiation (**a**) and irradiated with 120 eV hydrogen ions at fluences of 1.0 × 10^24^–1.5 × 10^25^/m^2^ (**b**–**k**), and its XRD spectrum (**l**).

The SEM observations of the polycrystalline W were performed at the same position when the hydrogen ion fluence varies from 1.0 × 10^24^ to 1.5 × 10^25^/m^2^, as shown in Fig. 3. Before the hydrogen ion irradiation, the polycrystalline W surface is very smooth, and the crystal boundaries are clearly identified (Fig. 3(a)). The periodic changes in the surface topography can be observed from Fig. 3(b–k). During the first period, the hydrogen ion fluence varies from 1.0 × 10^24^ to 4.0 × 10^24^/m^2^. After the hydrogen ion irradiation at a fluence of 1.0 × 10^24^/m^2^, grain boundaries are more clearly visible (Fig. 3(b)), indicating that surface sputtering resulting from hydrogen ion bombardment preferably occurs at the crystal boundaries. After the hydrogen ion irradiation at a fluence of 3.0 × 10^24^/m^2^, surface swelling is locally formed at crystal boundaries (Fig. 3(c)). When the hydrogen ion fluence increases to 4.0 × 10^24^/m^2^, the surface swelling diffuses into a large surface area (Fig. 3(d)). Surface microstructures are locally altered due to the hydrogen ion irradiation, indicating the formation of surface etching of polycrystalline W. After the hydrogen ion fluence further increases to 5.0 × 10^24^/m^2^, the fresh surface is formed, where no surface swelling and etching are visible (Fig. 3(e)).

The second periodic change (Fig. 3(e–g)) starts at a hydrogen ion fluence of 5.0 × 10^24^ ions/m^2^ while the third one (Fig. 3(h–k)) starts at a hydrogen ion fluence of ~9.0 × 10^24^ ions/m^2^. This shows the consistence with the periodic change in color of the irradiated W. During each period, the hydrogen ion irradiation initially results in the surface swelling at crystal boundaries, and the swelling diffuses into the whole surface area with increasing hydrogen ion fluence. The surface layer resulting from surface swelling is easily removed during the hydrogen ion irradiation. The XRD spectrum in Fig. 3(l) shows that the polycrystalline W is typically covered with a {200} or {100} crystal plane.

It has been reported that H can be trapped at or dissolved in W_2_C or WO_3_ to make intercalated compounds with H and further weakens the bonding^21^,^22^. The deposition of impurities, such as carbon and oxygen can alter the surface properties of irradiated tungsten, resulting in enhanced sputtering and transition of deposited species at the W surface^23^,^24^,^25^,^26^,^27^. Previous studies have indicated that tungsten erosion can be dominated by the impact of multiply charged low-Z impurity ions, such as C^4+^ 28,29,30. The multiply charged ions can obtain a relatively high energy when they travel through the plasma sheath and reach the W surface. However, different impurity ions can also change the material microstructure and change retention^31^. In this study, Raman spectroscopy and energy disperse spectroscopy (EDS) have been used to detect the surface microstructures and compositions of polycrystalline tungsten irradiated, and no impurities, such as C and O were found to exist on the W surface. The formation of W surface layer can be due to the low-energy hydrogen ion irradiation.

**Figure 4:**
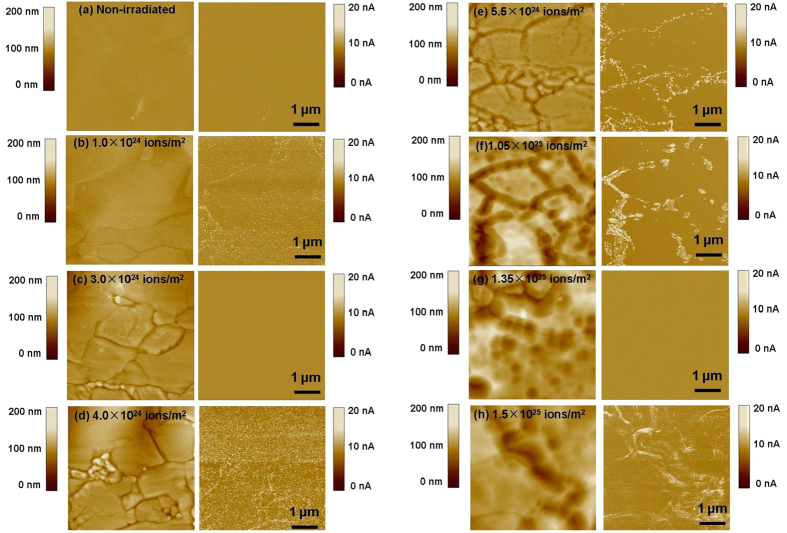
Surface topography (left) and simultaneously measured current images (right) of W crystal prior to H irradiation (**a**), and irradiated with hydrogen ions at fluences of (**b**) 1.0 × 10^24^/m^2^, (**c**) 3.0 × 10^24^/m^2^, (**d**) 4.0 × 10^24^/m^2^, (**e**) 5.5 × 10^24^/m^2^, (**f**) 1.35 × 10^25^/m^2^, and (**g**) 1.5 × 10^25^/m^2^. CAFM measurements were performed at *V*_tip_ = −20 mV (**b**–**g**).

Figure 4 shows the surface topography (left) and simultaneously measured current images (right) of the W (100) specimen prior to irradiation (a), and irradiated with the hydrogen ion fluence of (b) 1.0 × 10^24^/m^2^, (c) 3.0 × 10^24^/m^2^, (d) 4.0 × 10^24^/m^2^, (e) 5.5 × 10^24^/m^2^, (f) 1.05 × 10^25^/m^2^, (g) 1.35 × 10^25^/m^2^, and (h) 1.5 × 10^25^/m^2^. All CAFM measurements were performed at *V*_tip_ = −20 mV. The current image in Fig. 4(a) shows that low-density defects are already present prior to irradiation. These defects influence the local electron emission from the W surface. This current detection is very sensitive to a structural change in the surface layer, and these nanometer-sized defects are more conductive than other locations over the W surface. The defects can be generated in the surface layer during the mechanical polishing of W crystal.

After the W specimen was irradiated with a hydrogen ion fluence of 1.0 × 10^24^/m^2^, the density of defects is greatly improved (Fig. 4(b)). H atoms can get trapped into the vacancies, voids, or defects, leading to the growth of nanometer-sized defects in the surface layer^32^. The concentration of H atoms in the surface layer can be obviously increased due to the low-energy H irradiation. Our previous study has shown that the size and arrangement of defects are strongly dependent on the energy of the hydrogen ions and the W surface temperature^33^. Ordered arrangements of defects were formed at relatively high *E* and *T* because of stress-driven ripple effects of the defect growth at crystal grains.

After the W specimen was irradiated at a hydrogen ion fluence of 3.0 × 10^24^/m^2^, no obvious electron emission was observed from the current image (Fig. 4(c)). The dark color in Fig. 2 indicates the formation of a surface layer with different microstructures. The W surface becomes relatively insulating, which is presumably due to the existence of the surface layer. The surface layer can become loose or fall off, leading to an increase in the resistance of the surface layer measured. Indeed, CAFM measurements show that the W specimen in dark color becomes insulating after being irradiated at fluences of 5.5 × 10^24^/m^2^, 1.05 × 10^25^/m^2^, and 1.35 × 10^25^/m^2^, as shown in Fig. 4(e–g). This indicates that the surface layer generated with each period significantly influences the electron emission from the W surface. Our CAFM measurements show that the specimen in light color is conductive (Fig. 4(d,h)). High-density defects are formed due to the H irradiation. This indicates that after the surface layer was removed from W crystal, one fresh W surface is exposed to the hydrogen ions, where the hydrogen ion irradiation leads to the formation of nanometer-sized defects.

Figure 4(e,f) clearly show that the marginal zone of W surface layer is relatively conductive. This confirms that plenty of nanometer-sized defects are formed inside the W surface layer. The sputtering thresholds for H, D, and T on W are 477 eV, 209 eV, and 136 eV, respectively^34^, which is higher than the energy of 120 eV used in this study. The formation of nanometer-sized defects can decrease the thermal stability and the sputtering thresholds of W atoms in the surface layer, leading to its edge etching during the hydrogen ion irradiation at an energy of 120 eV. During low-energy hydrogen irradiation, W can be eroded by physical sputtering, and the sputtering threshold^35^ is about inversely proportional to the mass of the projectile and the surface binding of W.

**Figure 5:**
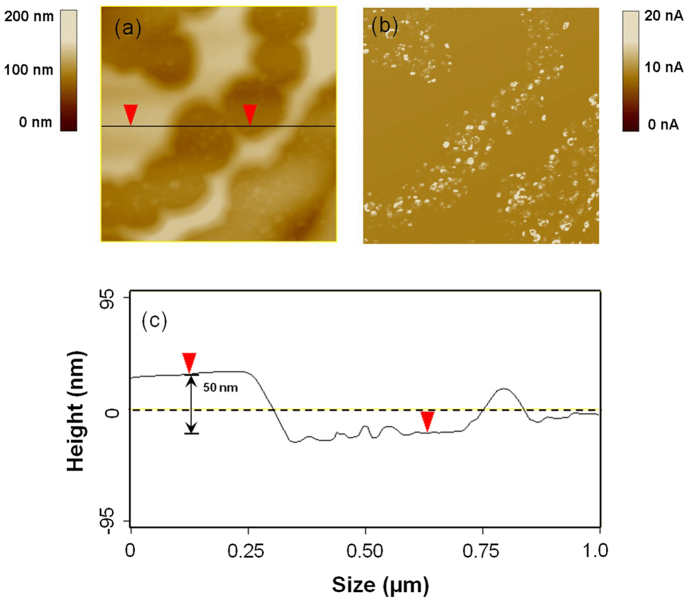
Surface topography (**a**), simultaneously measured current images (**b**), and cross-sectional view of surface topography (**c**) of the W crystal irradiated with a hydrogen fluence of 6.5 × 10^24^/m^2^. The current measurements were performed at *V*_tip_ = −20 mV.

Figure 5 shows the surface topography (a), the simultaneously measured current images (b), and a cross-sectional view of the surface topography (c) of the W (100) specimen irradiated with a hydrogen ion fluence of 6.5 × 10^24^/m^2^. Clearly, the W surface layer was locally etched because of the hydrogen ion irradiation. At some locations, the surface is delaminated, spalling can be observed and a fresh new surface is exposed. The surface layer is relatively insulating. After the surface layer being removed, the defects are formed due to H trapping into the W surface. The surface layer with loose structure was approximately 50 nm thick. This study indicates that the surface layer is easily etched during hydrogen ion irradiation, resulting in surface degeneration of the W crystal.

To evaluate the stability of W atoms on (100), we calculated the escape energy of W atoms from the 1^st^ and 2^nd^ layers of the W surface. According to the previous calculations^36^,^37^,^38^, the escape energy (*E*_*esp*_) for the W atom from the surface is defined as:


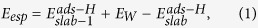


where 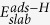 is the total energy of the W (100) surface with one H atom adsorbed and 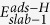 is the total energy of the surface with one W atom escaped from the 1^st^ or 2^nd^ layer of the (100) surface. *E*_*W*_ is the energy of an isolated W atom. According to the above definition, a positive value indicates an endothermic process. The *E*_*esp*_ represents the energy required for a W atom to escape from the surface. The lower the *E*_*esp*_, the more easily the W atom escapes from the surface.

The total energies for the W (100) surface with one H atom adsorbed at different positions are calculated and listed in Table 1. It is found that H is most energetically favorable to occupy site A (~2.32 Å to the surface) on the top of the 2^nd^ W atom. The escape energy calculated using Eq. 1 is also summarized in Table 1. The *E*_*esp*_ values for no H adsorption on surface are also listed in Table 1. Generally, the *E*_*esp*_ values of W atoms from the 1^st^ layer for H atoms situated at sites A, C, D are much lower than that of W atoms from 2^nd^ layer, with the exception of H occupying site B. H atoms positioned at inlayer of W (100) surface will diffuse spontaneously to the sites A, B, C, or D. The obtained escape energies in the 1^st^ and 2^nd^ layers with the presence of H adsorbed on a W (100) surface are somewhat decreased compared with the escape energy of W atoms from a clean W (100) surface. This simulation indicates that the stability of W atoms in the top layer is greatly decreased. If a high number of H atoms are adsorbed on the surface, the escape energy will be further decreased. The influence of H on the etching effect of the W surface may be qualitatively understood by: H absorbed on the W surface results in the escape of W atoms from the top layers, then W atoms in the inlayer will become the ones in the top layer exposed directly to H, which will then escape from the surface.

## Discussion

**Figure 6:**
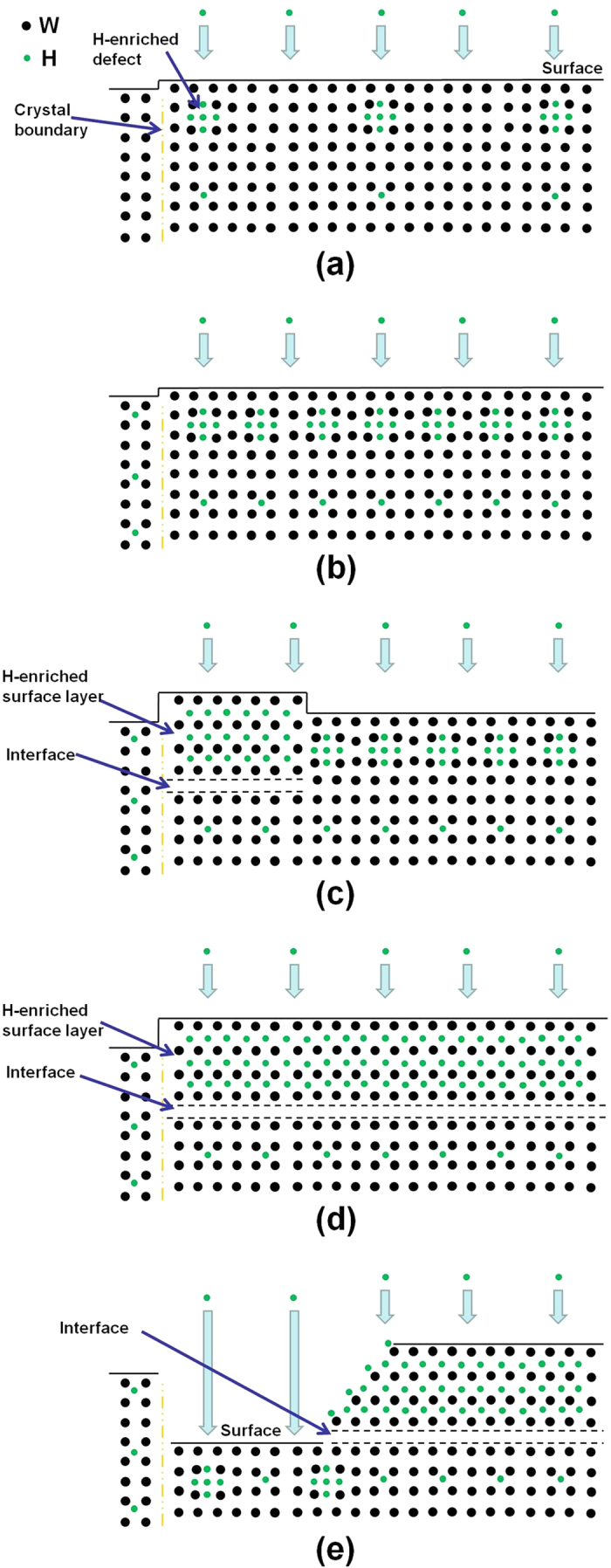
Schematic illustration of the periodic surface etching process due to low-energy hydrogen irradiation, where a H-enriched surface layer significantly results in the surface degeneration of the W (100) crystal. The periodic change includes the growth of nanometer-sized defects, and the formation, surface exfoliation, and etching of the W surface layer.

Depth profiles of hydrogen trapped in a W crystal irradiated with 200 eV hydrogen ions at 300–323 K have been determined up to a depth of 7 μm using the D(^3^He, p)^4^He reaction in a resonance-like technique^39^. The depth at which hydrogen is retained may be typically divided into three zones: (i) the near-surface layer (<100–200 nm), (ii) the sub-surface layer (from ~0.5 to ~2 μm), and (iii) the bulk (>5 μm). The H concentration at high ion fluences of ≥1 × 10^24^/m^2^ significantly decreases from several at.% in the near-surface layer to below 10^−3^–10^−4^ at% in the bulk. Our H irradiation conditions (hydrogen energy: 120 eV; temperature: 373 K; and hydrogen fluence: ≥1 × 10^24^/m^2^) are quite similar to the ones mentioned above. In our study, it was proposed that a H-enriched near-surface layer was formed during H irradiation. Indeed, the nanoindentation measurements were performed by Hystron nanohardness system (Hystron, TS-75), where the indentation depth varies in the range of 50–200 nm. Unlike high-energy heavy ion irradiation^40^, our measurements showed that the hydrogen ion irradiation results in a slight decrease in nanohardness from 7.7 to 6.6 GPa, indicating the effect of hydrogen penetration on the W surface. This is similar to the hydrogen embrittlement phenomenon^41^. Figure 6 depicts the layer-to-layer surface erosion process due to low-energy hydrogen ion irradiation, where the H-enriched near-surface layer significantly results in the surface degeneration of the W crystal.

After the energetic hydrogen species bombard the W surface, they can penetrate into the W crystal. The hydrogen atoms can diffuse along the surface layer, and a big number of hydrogen atoms get trapped into the vacancies and defects in the surface layer. Nanometer-sized defects are formed in the surface layer during H irradiation (Fig. 6(a)). The defects of the W crystal irradiated with low-energy hydrogen ions have been detected by the CAFM method. The inner stress in the surface layer is generated due to the growth of defects. With increasing H accumulation, the density of nanometer-sized defects is improved, leading to an obvious increase in the inner stress of the surface layer (Fig. 6(b)). The H concentration in the sub-surface layer is greatly improved during the H accumulation, resulting in a H-enriched surface layer. Due to its high inner stress, the H-enriched surface layer starts to expand and falls off at the grain boundaries (Fig. 6(c)), accompanied by the generation of an interface below the surface layer. Then surface exfoliation develops into the whole surface area of one W crystal grain (Fig. 6(d)). This inner stress of the H-enriched surface layer is rapidly released during the surface exfoliation, and plenty of H atoms inside the surface layer diffuse onto the surface in term of their thermal stability. This polycrystalline W with a H-enriched surface layer is in dark color, and the surface layer becomes relatively insulating, as detected by our CAFM.

Our DFT calculation confirms the thermal instability of W atoms in the top layer due to the adsorption of H atoms on the surface. H atoms adsorb on the W surface after they diffuse out of the H-enriched surface layer, which greatly decreases the thermal stability of the W surface layer. Surface etching occurs in the surface layer when hydrogen ions bombard the W surface (Fig. 6(e)). CAFM measurements confirm the existence of nanometer-sized defects in the W surface, which can lead to an obvious decrease in the sputtering threshold energy. The surface layer is easily removed during the surface etching. After the surface layer is etched, the fresh W surface is exposed to the hydrogen ions. With H accumulation, nanometer-sized defects are formed in the fresh W surface. Periodic surface etching of surface layers occurs during H irradiation, resulting in surface degeneration of the W crystal.

## Conclusions

The observation of a W crystal (100) irradiated with low-energy (120 eV) hydrogen ions by SEM and CCD camera shows the periodic change in the W surface microstructure. The hydrogen ion fluence per period was approximately 4 × 10^24^/m^2^. Measurements show the existence of a surface layer during H irradiation, which results in surface swelling and periodic changes in the surface color of the W crystal. CAFM measurements show that the surface layer is approximately 50 nm in thickness and relatively insulating. The W surface layer can contain a high concentration of hydrogen atoms, leading to an increase in its inner stress. Our finding suggests that the periodic surface degeneration of W crystal can be due to the lateral erosion of the W surface layer during the low-energy hydrogen irradiation. *E*_*esp*_ of the W atoms in the W (100) crystal is calculated by using the DFT method, and their thermal stability is compared. Our calculations confirm the thermal instability of W atoms in the top layer, especially in the case H atoms adsorb on the surface.

## Materials and Methods

The irradiated polycrystalline W specimens (Purity: 99.9 at.%) had the dimension of 15 × 12 × 0.8 mm. Their surfaces were mechanically mirror-polished to a surface RMS roughness of <10 nm. W specimens were annealed at 1273 ± 20 K for 2 h in vacuum with a background pressure of 10^−5^ Pa to relieve internal stresses and reduce the large concentration of nanometer-sized defects.

Previously, the materials irradiation experiment system (MIES) used for low-energy hydrogen irradiation has been described in detail elsewhere^42^,^43^. In this MIES, the vacuum chamber was pumped by a molecular pump (1600 L/s) with a mechanical pump (8 L/min). For all experiments, a base pressure of 3.0 × 10^−4^ Pa in the main chamber was achieved before the hydrogen ion irradiation. High-purified grade (>99.99%) hydrogen was introduced into the reactor through a mass flow controller. The H flow rate and pressure in the reactor were 100 standard cubic centimeters per minute (sccm) and 9 Pa, respectively. The 13.56 MHz RF plasma with a power of 400 W was used to generate an expanding plasma beam >50 mm in diameter with the ionic species of H^+^, H_2_^+^, and H_3_^+^ 44,45,46. W specimens were bombarded with hydrogen ions at normal incidence. The W surface 10 mm in diameter was exposed to the plasma beam during the irradiation. Measurements by a high-resolution optical emission spectrometry (OES, Princeton, SP-2750) show that the H_2_ plasma also contains H radicals and excited H_2_ molecules. No impurities, such as OH and CH were observed from OES measurements. A negative bias of −100 V was applied to the W specimen, which accelerated the H_X_^+^ (x = 1–3) ions in the plasma beam. The energy (*E*) of H_X_^+^ (x = 1–3) ions bombarding the W specimen was 120 eV when taking into account the plasma potential of 20 V. The ion current to the W specimen was about 10 mA, corresponding to a H_X_^+^ (x = 1–3) flux of ~1.0 × 10^20^ ions/m^2^·s. These irradiation conditions result in a W surface temperature of 373 ± 20 K. The surface temperature (*T*) of W specimens was measured with an infrared STL-150B pyrometer. In this study, the H_X_^+^ (x = 1–3) fluence was varied from 1.0 × 10^24^ to 1.5 × 10^25^/m^2^.

W specimen was marked prior to the hydrogen ion irradiation. The marked place was utilized for the observation of the irradiated specimen by scanning electron microscope (SEM, Hitachi S-4800). SEM measurements were performed after each irradiation step with hydrogen ion fluences of (0.5–3.0) × 10^24^/m^2^. The optical reflection images were also captured by a CCD camera.

In our previous studies^3^,^33^,^42^, CAFM (Veeco DI 3100) has been used to detect the nanometer-sized defects in the polycrystalline W irradiated with low-energy helium or hydrogen ions. From CAFM measurements, one can simultaneously obtain the surface topography and current image of an irradiated W specimen at a typical scan rate of 0.30 Hz. In the CAFM method, a laser system is used to keep a constant deflection of the PtIr-coated tip which is in contact with the specimen. During CAFM measurement, a constant voltage of −20 mV is applied between the conductive tip and the W specimen. When the conductive tip is scanned across the surface of the irradiated W specimen, the difference in the electron emission intensity across the surface is identified from the current measurement. The nanometer-sized defects, resulting from H implantation into the W surface layer can alter the electron emission through the AFM tip. The 2-dimensional distribution of the measured current yields a current image. This CAFM method is very sensitive to changes in the microstructure in the near-surface layer of polycrystalline W^3^,^42^. This method can be used to efficiently compare the surface microstructure with the distribution of defects in the near-surface layer, and it does not induce any damage to the measured specimen.

## Additional Information

**How to cite this article**: Fan, H. *et al.* Surface degeneration of W crystal irradiated with low-energy hydrogen ions. *Sci. Rep.* **6**, 23738; doi: 10.1038/srep23738 (2016).

## Figures and Tables

**Table 1:** 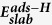
 , 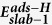 , and *E*_*esp*_ values of the (100) W slab with one H atom adsorbed on high-symmetry surface positions (A, B, C, and D).

H position	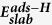	Position of escaped W	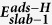	*E*_*esp*_
No H adsorption	–	layer 1	–	8.17
No H adsorption	–	layer 2	–	11.35
A	−552.47	layer 1	−540.00	8.10
A		layer 2	−536.64	11.47
B	−553.27	layer 1	−539.76	9.15
B		layer 2	−540.30	8.61
C	−552.53	layer 1	−540.29	7.88
C		layer 2	−537.52	10.65
D	−553.25	layer 1	−539.78	9.10
D		layer 2	−537.95	10.93

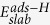
 is the total energy of the slab with the H atom adsorbed on A, B, C, or D positions. 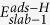 is the total energy of the slab with one W atom escaped from the surface layers 1 or 2. *E*_*esp*_ is the escape energy of W atoms in the surface layers 1 or 2.
